# *Burkholderia phytofirmans PsJN* reduces impact of freezing temperatures on photosynthesis in *Arabidopsis thaliana*

**DOI:** 10.3389/fpls.2015.00810

**Published:** 2015-10-02

**Authors:** Fan Su, Cédric Jacquard, Sandra Villaume, Jean Michel, Fanja Rabenoelina, Christophe Clément, Essaid A. Barka, Sandrine Dhondt-Cordelier, Nathalie Vaillant-Gaveau

**Affiliations:** ^1^Unité de Recherche Vignes et Vins de Champagne – EA 4707, Laboratoire de Stress, Défenses et Reproduction des Plantes, UFR Sciences Exactes et Naturelles, SFR Condorcet FR CNRS 3417, Université de Reims Champagne-ArdenneReims, France; ^2^Laboratoire de Recherche en Nanosciences, Pôle FarmanReims, France

**Keywords:** *Burkholderia phytofirmans PsJN*, cold, photosynthesis, PGPR, *Arabidopsis*

## Abstract

Several plant growth-promoting rhizobacteria (PGPR) are known to improve plant tolerance to multiple stresses, including low temperatures. However, mechanisms underlying this protection are still poorly understood. The aim of this study was to evaluate the role of the endophytic PGPR, *Burkholderia phytofirmans* strain *PsJN* (*Bp PsJN*), on *Arabidopsis thaliana* cold tolerance using photosynthesis parameters as physiological markers. Under standard conditions, our results indicated that *Bp PsJN* inoculation led to growth promotion of *Arabidopsis* plants without significant modification on photosynthesis parameters and chloroplast organization. However, bacterial colonization induced a cell wall strengthening in the mesophyll. Impact of inoculation modes (either on seeds or by soil irrigation) and their effects overnight at 0, -1, or -3°C, were investigated by following photosystem II (PSII) activity and gas exchanges. Following low temperatures stress, a decrease of photosynthesis parameters was observed. In addition, during three consecutive nights or days at -1°C, PSII activity was monitored. Pigment contents, RuBisCO protein abundance, expression of several genes including *RbcS, RbcL, CBF1, CBF2, CBF3, ICE1, COR15a*, and *COR78* were evaluated at the end of exposure. To assess the impact of the bacteria on cell ultrastructure under low temperatures, microscopic observations were achieved. Results indicated that freezing treatment induced significant changes in PSII activity as early as the first cold day, whereas the same impact on PSII activity was observed only during the third cold night. The significant effects conferred by *PsJN* were differential accumulation of pigments, and reduced expression of *RbcL* and *COR78*. Microscopical observations showed an alteration/disorganization in *A. thaliana* leaf mesophyll cells independently of the freezing treatments. The presence of bacteria during the three successive nights or days did not significantly improved *A. thaliana* responses but prevented the plasmalemma disruption under freezing stress.

## Introduction

Extreme environmental events such as prolonged drought, heavy rains or cold are likely to increase in the future due to climate change. Cold stress, including chilling (0∼10°C) and freezing (<0°C) temperatures, slows plant growth and development (Rohde et al., 2004; Miura and Furumoto, 2013), causes plant death (Ruelland et al., 2009) and thus reduces crop yield (Boyer, 1982; Nagarajan and Nagarajan, 2010). Plants percept cold and reply by multiple adjustments at physiological, biochemical, and molecular levels (Theocharis et al., 2012b). These include reduced membrane fluidity (Orvar et al., 2000), ultrastructural modifications in cell components, including plastids and mitochondria (Zhang et al., 2011), transiently increased cytosolic Ca^2+^ levels (Ruelland et al., 2009; Fanucchi et al., 2012), reprogramming of the transcriptome and the proteome (Thomashow, 1998; Chinnusamy et al., 2007; Miura and Furumoto, 2013). Plants sense low temperatures and activate multiple transcriptional cascades, one of which involves *CBF* and *ICE1*. *CBF* genes play important roles in cold acclimation and are regulated by multiple pathways (Thomashow, 2010). Three genes (*CBF1/DREB1b, CBF2/DREB1c*, and *CBF3/DREB1a*) have been well-studied in *Arabidopsis*. Their transcription is activated only a few minutes after transferring plants to low temperature (Gilmour et al., 2004; Medina et al., 2011) and is followed by induction of *CBF* target genes, such as *COR* genes (Svensson et al., 2006). COR proteins may protect cells against environmental chilling stress or regulate gene expression during the adaptive response (Fowler and Thomashow, 2002).

Chloroplasts are the main organelle impacted by cold and photosynthesis is one of the traits that are rapidly affected by cold (Kratsch and Wise, 2000; Theocharis et al., 2012b). Cold exposures might affect chloroplast ultrastructure by altering chlorophyll antenna complexes (Ensminger et al., 2006) or/and modifying thylakoid structures (Hincha and Schmitt, 1992; Adam and Murthy, 2014). The restricted photosynthetic processes by cold temperatures lead to a lack of plant energy resource (Ensminger et al., 2006; Biswal et al., 2011). Chilling temperatures also led to stomatal closure in many cold-tolerant plants such as *Arabidopsis* (Kozlowski and Pallardy, 1979; Cornic and Ghashghaie, 1991; Wilkinson et al., 2001; Rohde et al., 2004), but not in cold-sensitive plants (Wilson, 1976; Lee et al., 1993). Stomatal closure limited leaf dehydration (Davies et al., 1982), but restricted CO_2_ uptake, and thus reduced photosynthetic activity. The photosynthetic activity is due to the RuBisCO activity, enzyme able to fix carbon in the chloroplast. The protein is composed of two subunits: the nuclear-encoded *RbcS* gene and the chloroplast-encoded *RbcL* gene. Accordingly, enzymatic activities involved in sugar synthesis slowed down during cold exposure (Hurry et al., 2000). Further, eleven proteins involved in the photosynthetic apparatus of *Arabidopsis* are modulated by freezing conditions (Fanucchi et al., 2012). Among them, the oxygen-evolving enhancer protein 1–1 and the RuBisCO large chain significantly accumulated. In contrast to RuBisCO, other Calvin cycle enzymes (RuBisCO activase, phosphoglycerol kinase, glyceraldehyde-3-phosphate dehydrogenase, stromal fructose-1,6-bisphosphatase, ribulose-5-phosphate-3-epimerase, and phosphoribulokinase) showed significant reductions (Goulas et al., 2006). Moreover energy dissipation through non-photochemical quenching in cold condition could enhance cold acclimation and protect plants from oxidative damage (Ruelland and Zachowski, 2010). Furthermore, a concomitant rise in zeaxanthin levels was observed to protect the PSII reaction center from over-excitation (Król et al., 1999; Theocharis et al., 2012b). A reversible decrease of PSII activity by cold night was reported in grapevine inflorescence (Sawicki et al., 2012), *Arabidopsis* leaf (Zhang and Scheller, 2004), and tomato leaf (Liu et al., 2012). Moreover, light absorption decreases less than carbon fixation, which leads to generation of reactive oxygen species and thus, oxidative stress (Huner et al., 1998; Allen and Ort, 2001).

Plant tolerance to cold also depends on environmental regulators such as photoperiod and light quality (Thomashow, 1999; Kim et al., 2002). Wanner and Junttila (1999) have argued that light was required for cold acclimation in plants. These cold-adaptive processes impact photosynthesis mechanisms to re-establish cellular energy balance (Stitt and Hurry, 2002; Ensminger et al., 2006; Biswal et al., 2011).

Some bacterial strains of plant rhizosphere induced beneficial effect on plant growth (Kloepper et al., 1989, 2004; Glick, 1995; Mantelin and Touraine, 2004; Hayat et al., 2010). Such groups of bacteria are called PGPR and directly or indirectly promote plant growth by different mechanisms, e.g., phosphorus-solubilization, N_2_-fixation, uptake facilitation of some soil nutrients, phytohormone production (Mantelin and Touraine, 2004; Bhattacharyya and Jha, 2012). Effects of PGPR are highly dependent on the plant-bacteria interaction (van Loon, 2007). Zhang et al. (2008) found that PGPR strain *Bacillus subtilis* GB03 increased chlorophyll content and photosynthetic efficiency of *Arabidopsis* by modulation of endogenous glucose concentration and abscisic acid signaling. PGPR could also protect plants against biotic and abiotic stresses (Yang et al., 2009) by inducing plant physical, chemical, and genetic modifications (Grover et al., 2011). *Bp PsJN* improves low temperature tolerance of grapevine by regulating expression of cold genes and inducing accumulation of proline, phenolic compounds and modification of carbohydrate metabolism (Ait Barka et al., 2006; Fernandez et al., 2012b; Theocharis et al., 2012a). Ait Barka et al. (2006) showed that *Bp PsJN* enhanced photosynthetic capacity and increased soluble sugar concentrations after chilling treatment in grapevine plantlets. Furthermore, Fernandez et al. (2012a) specified that *Bp PsJN* caused a non-stomatal limitation of photosynthesis under cold conditions.

The aim of the present work was to analyze the effect of the PGPR *Bp PsJN* on photosynthetic-related responses of *Arabidopsis thaliana* to freezing stress. Firstly, impact of inoculation modes (either on seeds or by soil irrigation) and their effects overnight at 0, -1, or -3°C, was investigated by following PSII activity and gas exchanges. Secondly, plants were treated by three consecutive nights or days at -1°C. PSII activity was monitored in real time during all treatments. Pigment contents, RuBisCO protein abundance, expression of several genes including *RbcS, RbcL, CBF1, CBF2, CBF3, ICE1, COR15a*, and *COR78* were evaluated at the end of exposure. In parallel, the impact of *Bp PsJN* inoculation on the cell structure under freezing temperatures was displayed by transmission electronic microscopy.

## Materials and Methods

### Plant Material and Growth Conditions

All experiments were performed on wild type *A. thaliana* ecotype Columbia. Seeds were sown on soil. Plants were grown in a controlled environment chamber at 20°C/15°C (day/night), with 60% of relative humidity and a 12 h photoperiod (photosynthetically active radiation, PAR = 120 μmol m-^2^ s-^1^). For all of the experiments, measurements were performed on mature leaves of 5-weeks-old plants.

### Seed Bacterization

*Burkholderia phytofirmans* strain *PsJN* tagged with green fluorescent protein (Sessitsch et al., 2005) was grown for 24 h at 28°C at 180 rpm in King’s B liquid medium supplemented with kanamycin and cycloheximide (50 μg ml^-1^). Bacteria were collected after centrifugation at 4500 *g* for 10 min and suspended in phosphate-buffered saline (PBS, 10 mM, pH 7.2). To obtain bacterial inoculum of 5.10^8^ colony forming units per ml (cfu ml^-1^), the concentration was adjusted by spectrophotometry (OD 600 nm) to 0.5 (Pillay and Nowak, 1997). *Arabidopsis* seeds were immersed in bacterial inoculum of 5.10^8^ cfu ml^-1^ (SBp) or PBS for 3 h at 4°C (Mock).

### Cold Treatments during Three Consecutive Nights or Three Consecutive Days

Plants (Mock, SBp) were transferred to a cold growth chamber maintained at -1°C during 8 h of night-time (0–8 h) or 8 h of day-time (8–16 h) and at 20°C during the no freezing period. Control plants were kept at 20°C over the 3 days.

Immediately after the third night or day of cold treatment, leaves (Mock and SBp) were sampled in liquid nitrogen and stored at -80°C until use (Supplementary Figure S1). These samples were used to evaluate pigment contents and gene expression. Three independent biological replicates were performed (*n* = 9).

### Chlorophyll *a* Fluorescence

Photosystem II efficiency in *Arabidopsis* leaf was measured simultaneously during and after cold treatments with a chlorophyll fluorometer (MONITORING-PAM, Walz, Effeltrich, Germany). The MONITORING-PAM uses the repetitive saturation pulse method and provides an automatic data collection regime as described by Porcar-Castell et al. (2008). The pulses of light (1 s, 3500 μmol m^-2^ s^-1^) were applied every 20 min. Saturating pulse analysis detected and calculated fluorescence parameters of leaves automatically. Measurements were recorded with WinControl-3 software (Heinz Walz GmbH, Inc., Effeltrich, Germany). Three independent biological replicates were performed (*n* = 6).

### Pigment Quantification

Chlorophyll a and b, carotenoid pigments were extracted with acetone 80% and the concentrations were determined by spectrophotometry, according to the absorbance coefficients determined by Wellburn (1994).

### RNA Extraction and Real-Time PCR

RNA extraction and real-time PCR analysis were performed as described by Le Hénanff et al. (2013). For each sample, 100 mg of leaves were ground in liquid nitrogen. Total RNA was isolated using Extract’All (Eurobio), and 1 μg was used by reverse transcription using the Verso cDNA Synthesis Kit (Thermo Scientific) according to the manufacturer’s instructions. The transcript levels were determined by qPCR using the CFX 96^TM^ Real Time System (Biorad, France) and the SYBR Green Master Mix PCR kit as recommended by the manufacturer (Applied Biosystems). PCR conditions were 95°C for 15 s (denaturation) and 60°C for 1 min (annealing/extension) for 40 cycles. Traditional reference genes were evaluated with Bio-Rad CFX MANAGER software v.3.0 (*Actin2, UBQ5, UBQ10, EF1α*, and *Tubulin2*) to select a reference gene with a stable expression in all tested conditions (Hong et al., 2010). The expression stability geNorm M value of *UBQ5* was below the critical value of 0.5 and among the lowest under stress conditions. Transcript level was calculated using the standard curve method and normalized against *UBQ5* gene as an internal control. The specific primers used in this study were listed in Supplementary Table S1.

### Proteins Extraction and Western Blotting Analysis

Total proteins were extracted from 0.2 g of leaf with 500 μL cold extraction buffer (250 mM sorbitol, 50 mM Tris-HCl, pH 8.0, 2 mM EDTA, 7 g l^-1^ PVPP, 5 mM DTT, 1 mM PMSF, and 1/100 Halt Protease Inhibitor Cocktail-Thermo Scientific) and centrifuged at 10000 *g* for 10 min at 4°C. The supernatant was then collected and proteins were quantified by the Bradford method using bovine serum albumin as the standard (Bradford, 1976). Protein samples (2 μg) were solubilized for 3 min at 95°C in Laemmli buffer (Laemmli, 1970) and separated by SDS-PAGE in 12% (w/v) polyacrylamide gels, using Mini-protean three Cell electrophoresis equipment (Bio-Rad). Proteins were electro-transferred onto a polyvinylidene difluoride (PVDF) membrane using iBlot system (Invitrogen). Western blotting was performed according to standard procedures using rabbit anti-RbcL or -RbcS antibodies (Agrisera; 1:10000) and peroxidase-coupled anti-rabbit IgG antibodies (Cell signaling; 1:5000). Actin (Agrisera; 1:1000) was used as internal quantification control.

### Transmission Electron Microscopy of *Arabidopsis* Leaf Cell Structure

Fresh leaves were collected after a treatment of three consecutive nights or three consecutive days. They were prepared for microscopy analysis. Samples were fixed in 1% glutaraldehyde (0.1 M phosphate buffer, v/v) at pH 7.2 in 0.5% sucrose (w/v) and 0.2% Tween 20 (v/v) for 24 h and agitated at room temperature. After three rinses (5 min) in buffer, the leaves were post-fixed with 1% osmium tetroxide (w/v) in the buffer for 4 h. Leaves were then rinsed three times (5 min) in the buffer, dehydrated in an alcohol series, transferred to acetone and embedded in Araldite. Transverse ultrathin sections (80 nm nominal thickness) were cut (Reichert Jung Ultracut E) from the Araldite-embedded block and mounted on 200 mesh copper grids. Sections were observed under a JEM2100F TEM (JEOL) without post-staining. Micrographs were recorded using an Orius 200D CCD camera (Gatan). For each stage, 10 leaves from five plants were used.

### Statistical Analysis

Mann and Whitney test was used for all experiments, except for data generated by monitoring-PAM, which were analyzed by Student’s test. A repeated two way analysis of variance (ANOVA) was added for data of gas exchanges and PSII activity 2 h after one cold night.

## Results

The PGPR *Bp PsJN* is able to promote growth of several plants (Frommel et al., 1991; Nowak et al., 1995; Ait Barka et al., 2000; Poupin et al., 2013). In *A. thaliana, Bp PsJN* established root endophytic population independently of inoculation method (around 10^4^ cfu g-^1^ FW), whereas, no bacteria were detected in leaves (Supplementary Figure S2). This presence in roots triggered a significant promotion of aerial plant growth compared with the mock-treated plants (127, 126, and 128%, respectively; Supplementary Figure S3).

### Modification of Photosynthesis during and after a Cold Night

In order to understand if bacterial colonization and growth promotion may help plant to resist to low temperatures Photosynthetic parameters were investigated. Results showed that Fv/Fm of control plants decreased during the night (from 0.817 to 0.737), with a more pronounced effect during the cold night (Supplementary Figure S4A). However, the level of this decrease was dependent on the cold intensity. At 0 and -1°C, Fv/Fm was gradually reduced during the night. Despite that applied cold temperatures were reached after 30 min of exposure (Supplementary Figure S4B), the shift of photosynthetical response was recorded only 60 min later in leaves treated at -3°C. The endophytic presence of *Bp PsJN* in *A. thaliana* has no impact on plant response to cold.

Two-way analysis of variance revealed a negative impact of cold temperatures on all tested photosynthetic parameters (Supplementary Table S2). Compared to 15°C, exposure at 0 or -1°C did not modified the ΦPSII on *Arabidopsis* plants (Supplementary Figure S5A). After one night at -3°C, ΦPSII was only weakened in *Bp* plants. In addition, Pn was reduced for all three cold treatments, with more impact at -3°C than at 0 or -1°C (Supplementary Figure S5B, Supplementary Table S2). The presence of *Bp PsJN* (SBp and SBp+Bp) increased the Pn after the cold night at -3°C. For the Mock plants, intercellular CO_2_ concentration decreased after the cold night at 0 or -1°C, but not at -3°C (Supplementary Figure S5C). Considering the Pn reduction by cold stress, these results suggested that 0 and -1°C treatments triggered stomatal limitation to Pn on *Arabidopsis*, but -3°C treatment led to a non-stomatal limitation. *Bp PsJN* inoculations only affected Ci after one night at -3°C: Bp and SBp+Bp plants displayed a lower Ci compared with Mock and SBp plants. The three cold temperatures triggered a similar decrease of g_s_ (Supplementary Figure S5D) and E (Supplementary Figure S5E) without impact of bacterial inoculation (Supplementary Table S2). According to reported results, the seed bacterization was selected for further investigation. In addition, only treatment at -1°C was chosen since -3°C was too low to be applied during three consecutive days.

### Impact of Three Consecutive Nights or Days at -1°C

#### PSII Activity

**FIGURE 1:**
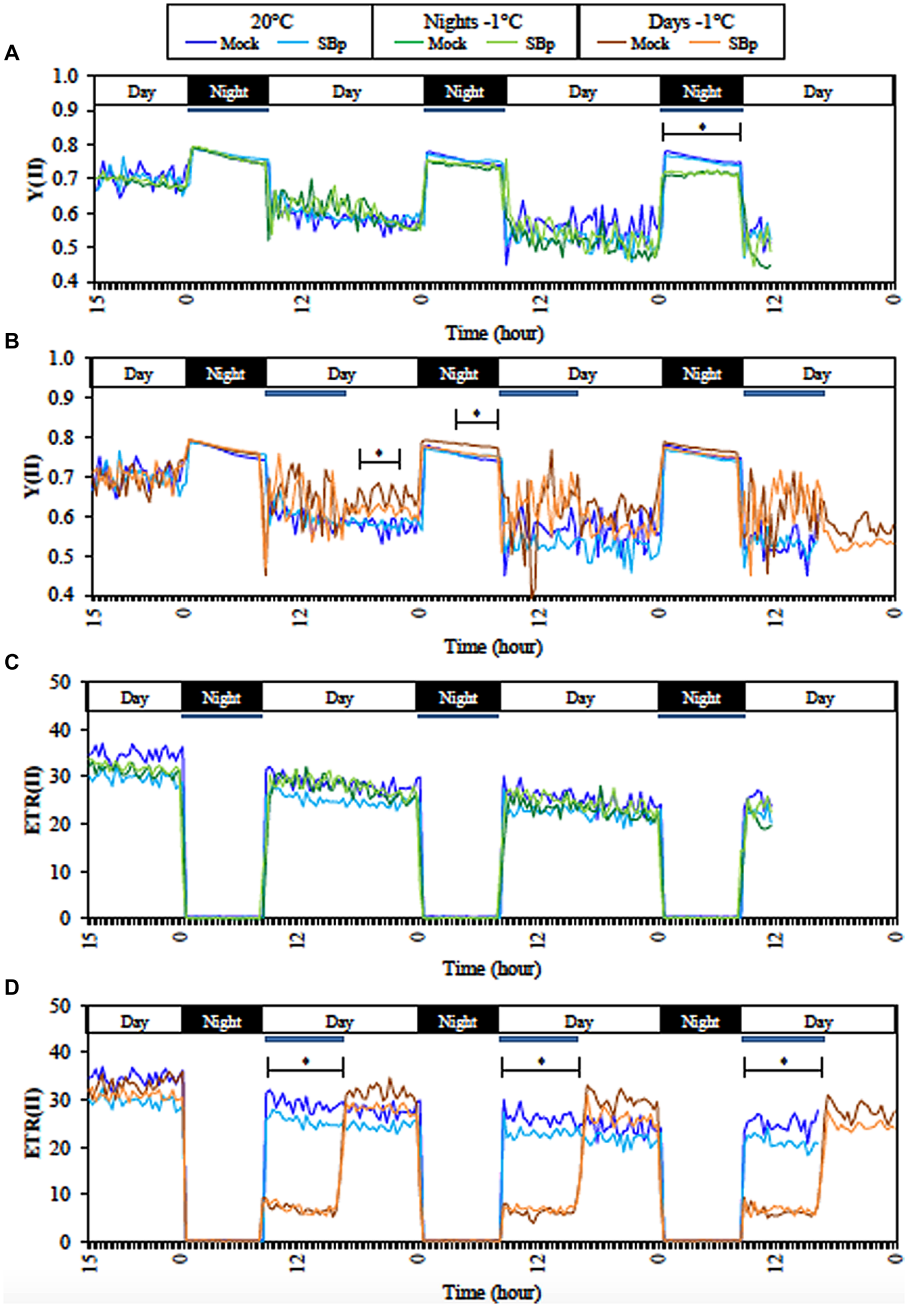
**Continuous measurements of fluorescence parameter during treatments with Monitoring PAM.** Real-time efficiency of PSII (YII) during three consecutive nights **(A)** or days **(B)** at -1°C and at 20°C. Real-time electron transport rate (ETRII) of PSII during three consecutive nights **(C)** or days **(D)** at -1°C, and at 20°C. Data are averages of three independent experimental replicates, each with two plants per treatment (*n* = 6). Blue bars represent cold period (-1**°**C). No significant differences between Mock and SBp conditions was observed; asterisks (^∗^) show the period of significant differences between cold and control conditions (20°C; Student’s test, *P* < 0.05).

Non-destructive continuous measurements of chlorophyll *a* fluorescence were thus used as a sensitive indicator of photosynthetic performance during night treatments. The monitoring-PAM was used to follow quantum yield of PSII (YII) and ETRII each 20 min during three consecutive cold-nights or cold-days (**Figure 1**). During the day, YII measurements reflect the ΦPSII and during the night, the Fv/Fm. The first and the second cold night did not affect Fv/Fm, but it was significantly reduced by cold during the third night. Moreover, ΦPSII was not affected by cold nights (**Figure 1A**). However, ΦPSII and Fv/Fm were significantly increased after the first cold day. But this increase was not significant after the second or third cold day of treatment (**Figure 1B**). Neither ΦPSII nor Fv/Fm was affected by the presence of *Bp PsJN* during the three consecutive nights or days (**Figures 1A,B**).

Electron transport rate II (ETRII) was not affected by the three consecutive nights at -1°C, whereas it decreased during the cold days. However, ETRII came back to similar level to control plant immediately after the end of cold treatment (**Figures 1C,D**). *Bp PsJN* colonization did not affect ETRII.

#### Pigment Contents

The content of photosynthetic pigments was analyzed to clarify the observed variations in the chlorophyll fluorescence.

**FIGURE 2:**
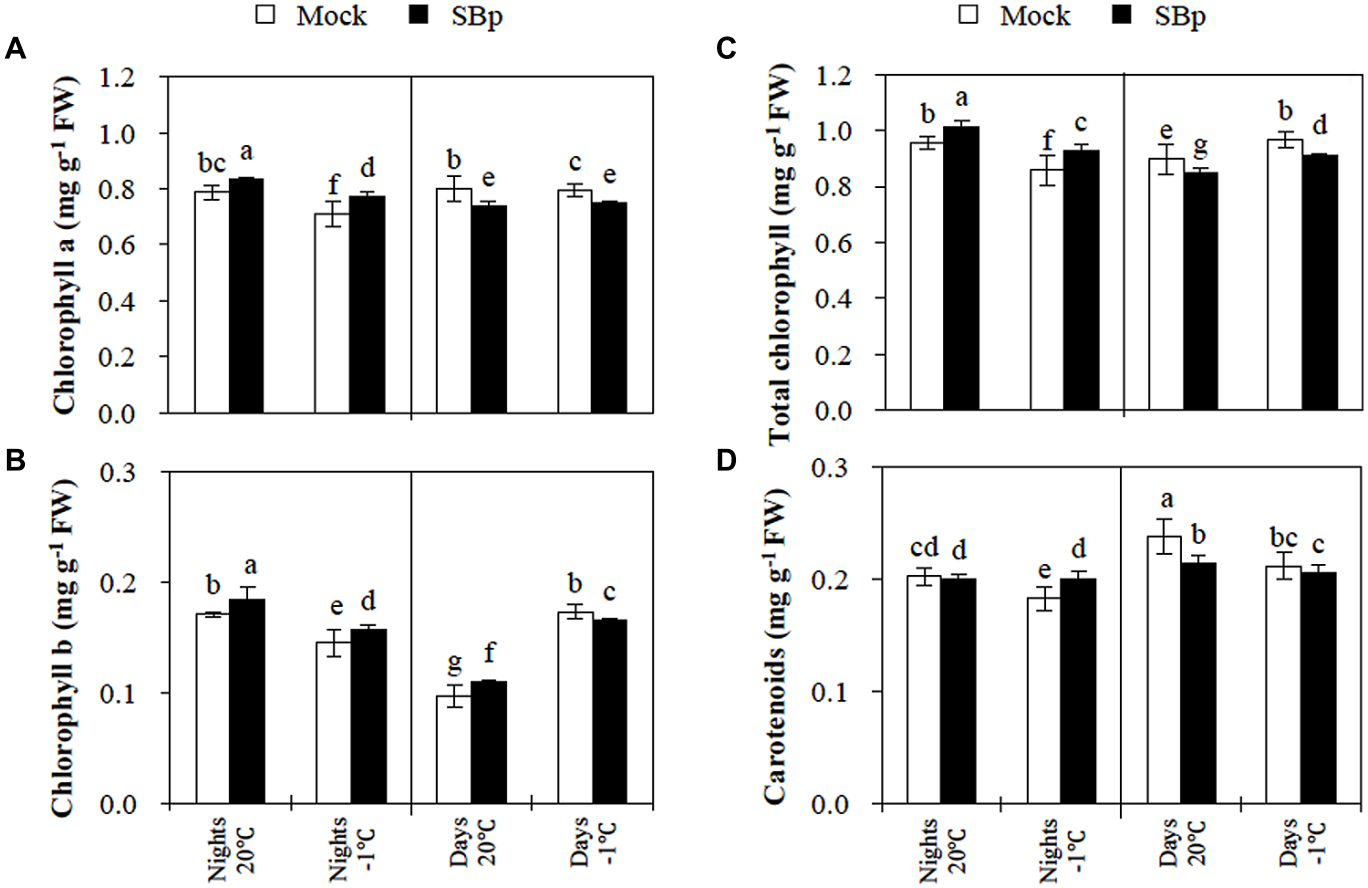
**Pigment concentration in *Arabidopsis* leaves of Mock and bacterized plants (SBp) after three consecutive nights or days at -1**°**C. (A)** Chlorophyll a, **(B)** chlorophyll b, **(C)** total chlorophyll, and **(D)** carotenoids concentration. Data (mean ± SE) are averages of three independent experimental replicates, each with three plants per treatment (*n* = 9). Same letters indicate non-significant differences among all conditions (Mann–Whitney test; *P* < 0.05).

After three nights at -1°C, the results report that chlorophyll (a and b) and carotenoid contents decreased compared to non-chilled plants (**Figure 2**). The presence of *Bp PsJN* induced an accumulation of both chlorophylls (a and b) whereas carotenoid levels were not modified independently of cold treatment.

After 3 days at -1°C, total chlorophyll content increased, due to an accumulation of chlorophyll b (**Figures 2A–C**). However, the carotenoid levels decreased in response to the cold stress (**Figure 2D**). The presence of *Bp PsJN* triggered a reduction in total chlorophyll content, mainly due to a decrease in chlorophyll a (**Figures 2A–C**). Similarly, in bacterized plants, carotenoid levels slightly decreased at 20°C as well as at -1°C (**Figure 2D**).

#### Modifications of Leaf Mesophyll Cell Ultrastructure

**FIGURE 3:**
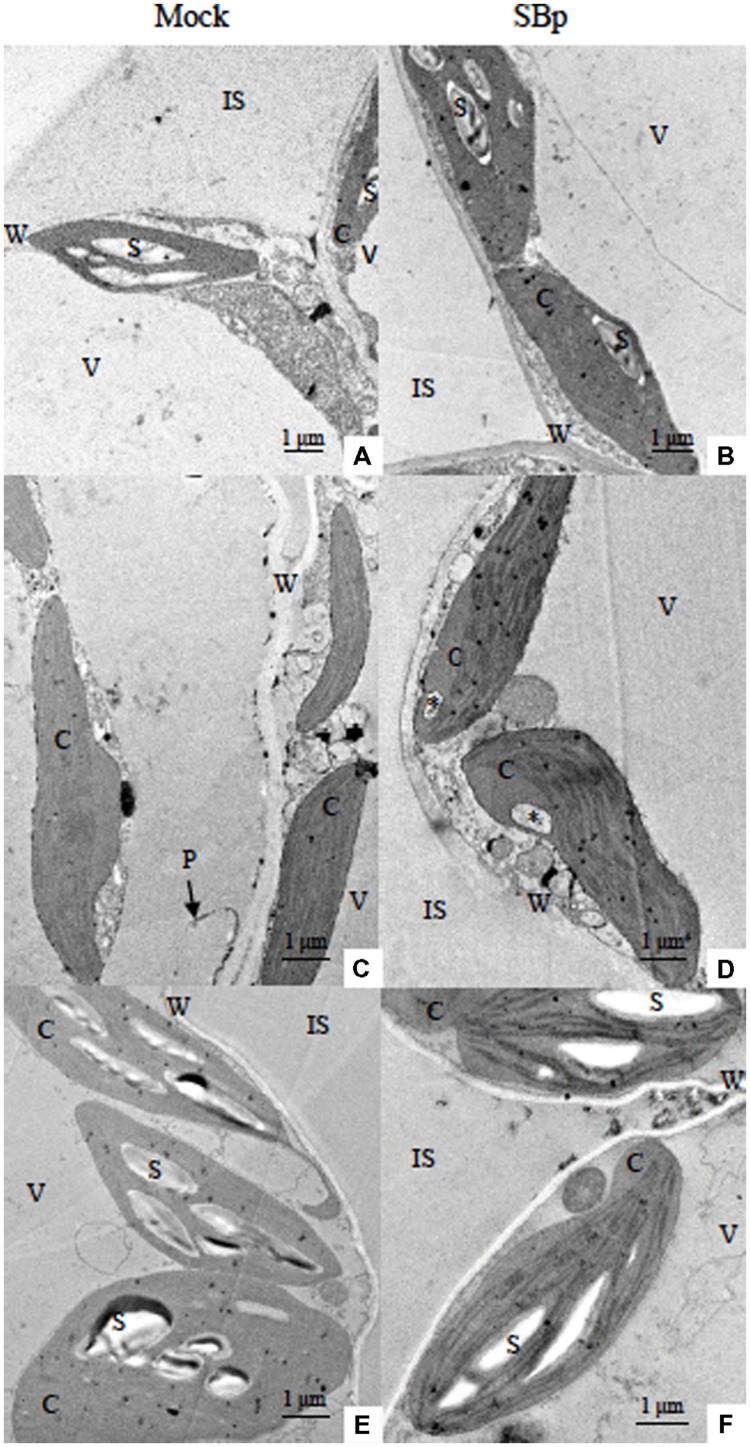
**Transmission electron micrographs of *Arabidopsis* leaf mesophyll cells after three consecutive nights or days at -1°C.** Images shown the leaf mesophyll cells of Mock **(A,C,E)** or bacterized plants (SBp; **B,D,F)** grown under control conditions (20°C; **A,B)**, cold nights **(C,D)** or cold days **(E,F)**. ^∗^Invagination open to cytoplasm near one end of a chloroplast; C, chloroplast; IS, intercellular spaces; P, plasmalemma; S, starch; V, vacuole; W, cell wall.

The major adverse effect of cold stress in plants has been seen in terms of plasma membrane damage. Such changes induced by cold stress adversely affect the growth and development of plants. In this study, modifications of leaf mesophyll cell ultrastructure were observed to illustrate impact of cold stress. TEM observations showed an alteration/disorganization in *Arabidopsis* leaf mesophyll cells following freezing treatments in bacterized or control plants. The control plants (20°C, Mock) exhibited organized cells (**Figure 3A**). Chloroplasts displayed lens-like oblong shapes, exhibited starch granules and were lined close to the plasmalemma (**Figure 3A**). The cold stress induced modifications of cell organization and chloroplast ultrastructure (**Figures 3C** or E). Both freezing treatments induced a cell wall strengthening (**Figures 3C,E**; **Table 1**). Although freezing stress during the day did not impact the granule starch content (**Figures 3E,F**), the cold-stressed plants under night condition presented chloroplast without starch granule whatever the presence of *Bp PsJN* (**Figures 3C,D**). Finally, some cells of stressed-plants during the night, presented a disruption of plasmalemma leading to a cell disorganization (chloroplasts and cytoplasm distant from the cell wall due to plasmalemma disruption; **Figure 3C**). Indeed, while no plamalemma disruption was observed in control plants, 45.8 and 48.6% of mesophyll cells exhibited disrupted membrane when cold was applied during 3 days or three nights respectively (**Figure 3C**; **Table 2**).

**Table 1:** Impact of three consecutive nights or days at -1°C, or bacterial treatments on cell wall thickness with results from Mann–Whitney test.

Treatments	20°C	Nights-1°C	Days-1°C
Mock	0.13 ± 0.04b	0.21 ± 0.06a	0.19 ± 0.04a
SBp	0.20 ± 0.05a	0.23 ± 0.07a	0.19 ± 0.03a

*Data (mean ± SE) are average of thickness of cell wall in microns (*n* = 15). Same letters indicate non-significant differences among all conditions (Mann–Whitney test; *P* < 0.05).*

**Table 2:** Percentage of plamalemma disruption according to growth conditions in Mock and bacterized plants (SBp).

Treatments	20°C	Nights-1°C	Days-1°C
Mock	0c	45.8 ± 11.2a	48.6 ± 11.7a
SBp	0c	17.9 ± 10.9b	12.1 ± 14.1b

*The percentages of disrupted membranes (data ± SE) were evaluated among a total of 30 mesophyll cells.*

*Same letters indicate non-significant differences among all conditions (Mann–Whitney test; *P* < 0.05).*

The TEM observations did not allowed to observe the presence of bacteria in the leaf mesophyll. At 20°C, *Bp PsJN* inoculation did not modify the chloroplast organization or the presence of starch granule (**Figure 3B**). Interestingly, bacterized plants presented cells with strengthened cell wall compared to control plants (**Table 1**). The cell wall thickness of bacterized plants was similar to cold stressed plant (**Figures 3B–F**; **Table 1**). The *Bp PsJN* inoculation seemed to prevent plasmalemma disruption under freezing stress by night (**Figure 3D**). Indeed, cells with disturbed plasmalemma was approximately 2.5 times less abundant in bacterized-plants compared to non-bacterized plants under cold-treatment (**Table 2**). Nevertheless, the bacterization did not seem to impact the other effects of cold stress (**Figures 3C–F**).

#### Regulation of RuBisCO

**FIGURE 4:**
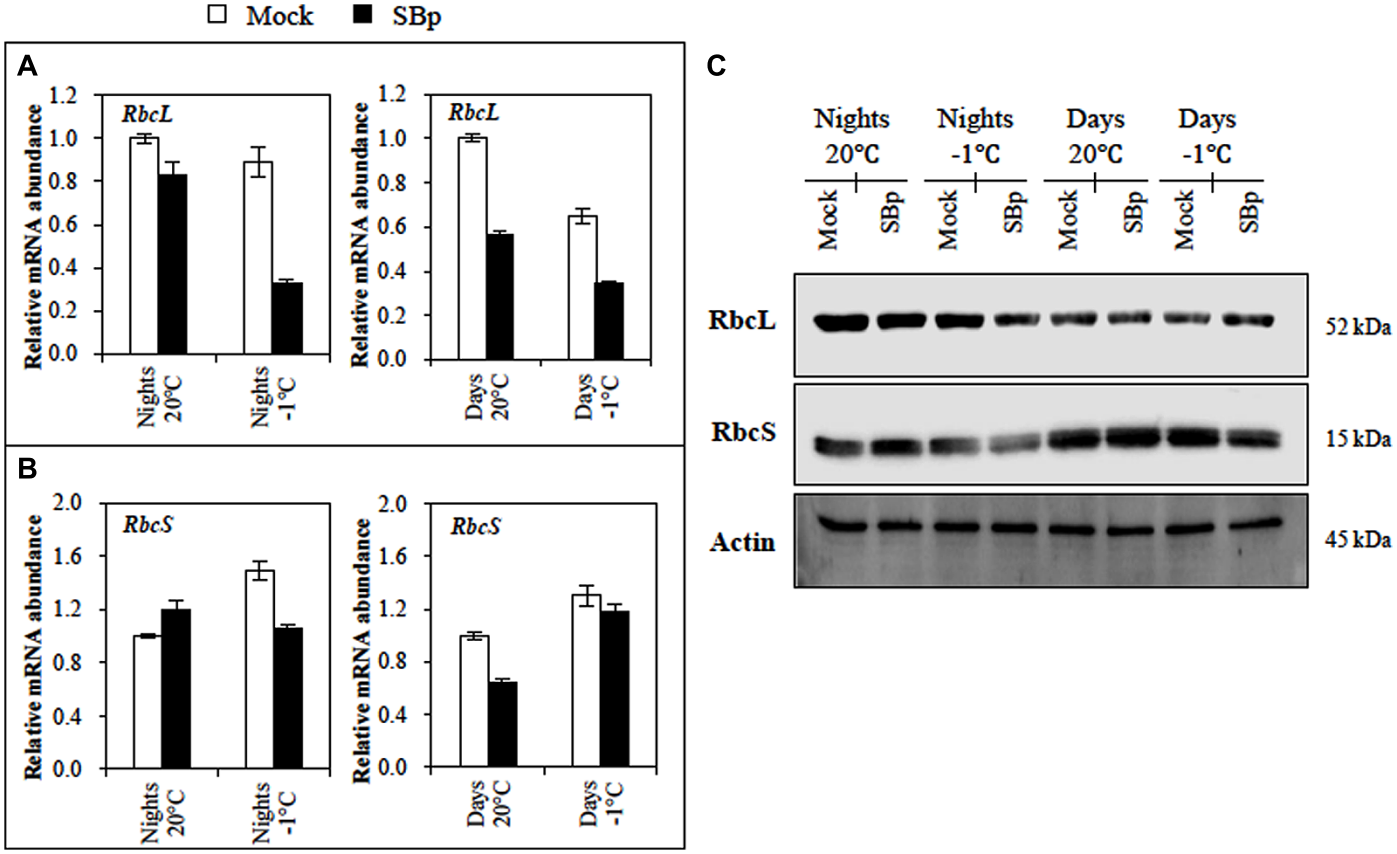
**RuBisCO regulation in *Arabidopsis* leaves of Mock and bacterized plants (SBp) after three consecutive nights or days at -1°C.** Gene expression levels of RuBisCO subunit: **(A)** *RbcL* and **(B)** *RbcS*. Data (mean ± SE) represent mean fold increases in transcript level relative to those of control plants (Mock, maintained at 20°C) in one representative experiment among three independent repetitions. **(C)** Western blots for RbcL and RbcS. Normalization was carried out with Actin. Numbers on the right indicate molecular mass in kilodaltons. Data display the results of one representative experiment among three independent repetitions.

To explore whether the difference in photosynthetic activity could be due to the RuBisCO, expression of genes encoding the two subunits of the RuBisCO (the nuclear-encoded *RbcS* and the chloroplast-encoded *RbcL*) and their protein accumulation were followed. Our results showed that neither cold nor inoculation with *Bp PsJN* modified *RbcS* expression. However, RbcS accumulation was less pronounced in bacterized plants exposed to -1°C during three consecutive nights (**Figures 4B,C**). For RbcL, a stronger accumulation was observed when plants were exposed to cold during the night than during the day. Bacterized plants also showed a significant reduction of *RbcL* expression, either at 20°C or at -1°C but such reduction in protein accumulation was only visible upon cold night exposure (**Figures 4A,C**).

#### Regulation of the CBF Cold Response Pathway

**FIGURE 5:**
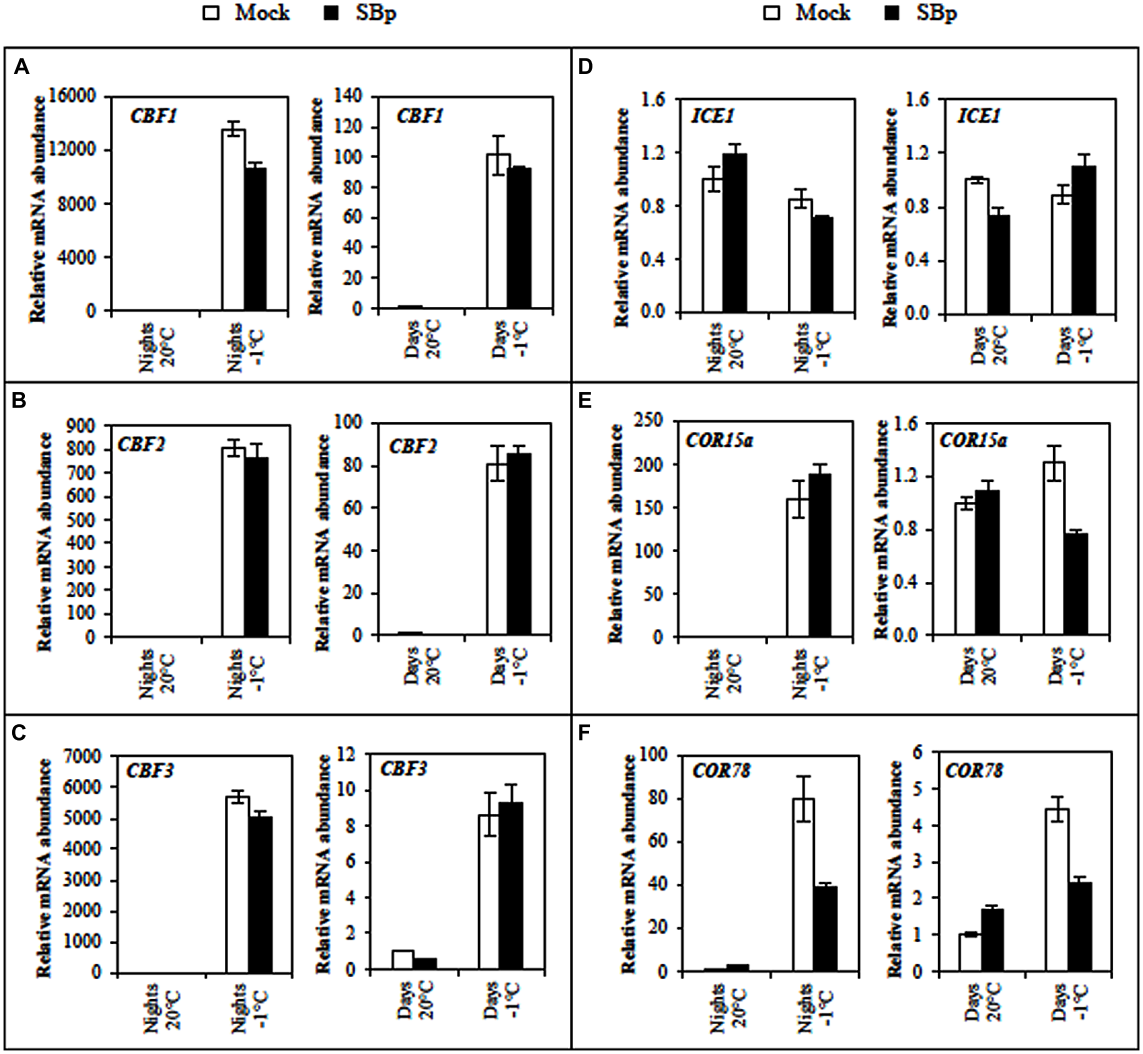
**Expression levels of the cold-induced genes **(A)***CBF1*, **(B)***CBF2*, **(C)***CBF3*, **(D)***ICE1*, **(E)***COR15a*, and **(F)***COR78* in *Arabidopsis* leaves of Mock and bacterized plants (SBp) after three consecutive nights or days at -1°C.** Data represent mean fold increases in mRNA levels relative to those of control plants (Mock, maintained at 20°C), referred to as the ×1 expression level. Values shown are mean ± SE of duplicate data from one representative experiment among three independent repetitions.

To explore whether the difference in photosynthetic activity could be correlated to a differential cold perception, cold-related marker gene expressions were analyzed before and after night or day cold stress. Expression pattern of cold genes (*ICE1, CBFs, COR15a*, and *COR78*) was monitored in mock- and *Bp PsJN*-inoculated plants. All these genes belong to the CBF cold response pathway, which contribute to freezing tolerance (Thomashow, 2010). With the exception of *ICE1*, all tested genes were induced by cold treatment (**Figure 5**). Curiously, induction levels were higher when cold was applied during the night than during the day. Both *COR* genes expression were repressed during cold day treatment, but only expression of *COR78* gene was repressed during cold night treatment in *Bp PsJN*-inoculated plants compared with non-inoculated ones (**Figures 5E,F**).

## Discussion

Plant growth-promoting rhizobacterias are known to induce several beneficial effects on plant growth and resistance to abiotic stresses. However, mechanisms underlying this interaction are currently poorly understood. The aim of this study was to better understand how the presence of a PGPR might influence *A. thaliana* photosynthetic mechanisms in response to cold temperatures and thus contribute to cold acclimation.

### Consequences of *Bp PsJN* Colonization on Plant Physiology and Cell Morphology

Our results showed that *Bp PsJN* was able to colonize *A. thaliana* roots and also to increase the plant aerial biomass. The colonization was limited to roots systems since no bacteria were detected in *Arabidopsis* leaves under our conditions. In contrast, Poupin et al. (2013) mentioned that *Bp PsJN* might be present in aerial organ of *A. thaliana*. This discrepancy might be due probably to the different ways of bacterial inoculation and plant growth conditions.

In standard conditions, when *Bp PsJN* was applied either on seeds or by soil irrigation, PSII activity, gas exchanges and RuBisCO (gene expression and protein content) were not affected. Considering the crucial role of RuBisCO in carbon fixation, this result was correlated with the unchanged Pn value. However, Ait Barka et al. (2006) have reported that *Bp PsJN*-inoculated grapevine plantlets exhibit a higher photosynthetic activity compared to non-bacterized plantlets. Further, switchgrass inoculation with *Bp PsJN* lead to an increased photosynthetic rate without change in g_s_ (Wang et al., 2015). In maize, *Bp PsJN* inoculation increased g_s_, photosynthetic rate, photochemical efficiency of PSII and also total chlorophyll contents (Naveed et al., 2014). In *Arabidopsis*, enhanced plant photosynthetic capacity by *B. subtilis* (Zhang et al., 2008) or *Bp PsJN* (Poupin et al., 2013) was also associated with an enhanced chlorophyll contents. However, in *Bp PsJN*-colonized grapevine plantlets, the decrease of chlorophyll contents was not associated with a ΦPSII variation (Fernandez et al., 2012a). In our case, chlorophyll (a, b and total) contents were increased by *Bp PsJN* inoculation in the non-stressed leaves sampled at 8 h, but this beneficial effect was not maintained when plants were sampled at 16 h. Differences between the two results may be related to plant circadian rhythm, which influence transcript abundance of genes associated with chlorophyll synthesis, heme production, chlorophyll accumulation, and synthesis of chlorophyll binding proteins (Dodd et al., 2005).

In the present study, *Bp PsJN* inoculation induced a cell wall strengthening in *A. thaliana* leaf. By their presence in plant tissues, bacteria are known to induce defence mechanisms of host plants (for review: Rosenblueth and Martinez-Romero, 2006). Among the plant defence responses, the strengthening of cell walls or the establishment of surrounding material inside the xylem or the cortex have been reported (Benhamou et al., 1998, 2000; James et al., 2002; Compant et al., 2005; Miché et al., 2006). Also, Frommel et al. (1991) showed an increase of the total plant lignin content in *Bp PsJN*-inoculated potato plantlets. Moreover, in grapevine plantlets, it was shown that *Bp PsJN* inoculation increases the cell wall thickness (Ait Barka et al., 2006) confirming thus our results showing that *Bp PsJN* reinforce the host cell wall.

### Consequences of Cold Stress

We showed that when the stress is timely short (one night), only a temperature of -3°C decreased Fv/Fm. Fv/Fm values reflect the degree of photoinhibition (Groom et al., 1991; Krivosheeva et al., 1996). When a moderate stress (-1°C) was applied, three cold nights were requested to trigger photosynthesis modifications, whereas only one cold day was sufficient to induce the same modifications. A reduced Fv/Fm level by cold nights has also been reported in *A. thaliana* (Zhang and Scheller, 2004) and other species (Boorse et al., 1998; Hendrickson et al., 2004; Oliveira and Peñuelas, 2004; Garstka et al., 2007; Liu et al., 2012). Cold stress led to the adjustment between the capacity to harvest light energy and the capacity to consume this energy into metabolic activity in leaves (Öquist and Huner, 2003). Increases in the ratio of electron transport to CO_2_ assimilation have been reported in leaves under chilling conditions (Fryer et al., 1998). In accordance with Kratsch and Wise (2000), our data showed that light combined with cold temperatures may cause greater damages, as the combination can disrupt photosynthesis by photoinhibition of PSII (increase in YII and decrease in ETR). Altogether, these data imply that light greatly exacerbates chilling injury due to energy overload.

Transmission electron microscopy observations showed that freezing induced chloroplast ultrastructure disorganization, inducing large part of stroma without thylakoid. Cold damage on photosynthetic apparatus could be due to reduced light-harvesting chlorophyll contents (Król et al., 1999) and decreased functional PSII reaction centers (Ensminger et al., 2006; Savitch et al., 2010). Huner et al. (1998) showed that cold stress modified biophysical properties of thylakoid lipids causing an increase in membrane viscosity, eventually resulting in inhibition of electron transport. Following cold treatment, a plasmalemma disruption was observed leading to the cell disorganization in leaf mesophyll. This phenomenon might be due to ice formation leading to cell dehydration and may result in cell collapse (Stefanowska et al., 1999). Likewise, the enlargement of ice crystals, due to freezing stress, could generate a mechanical constraint on the cell wall and plasma membrane, leading to the cell rupture (Pearce, 1999; Mahajan and Tuteja, 2005).

The chlorophyll reduction could be associated to environmental stress responses (Hendry and Price, 1993). In parallel, increase of carotenoid is frequently related with protection mechanisms against low temperatures by avoiding photoinhibition (Demmig-Adams, 1990; Hendrickson et al., 2004). According to our results, chlorophyll and carotenoid contents decreased after three nights at -1°C. However, while carotenoid levels were reduced after 3 days at -1°C, total chlorophyll was stimulated due to a slight increase in chlorophyll b. The observed decrease in photosynthetic pigment contents combined with disturbed chloroplast structures caused by freezing exposures might explain the reduction of PSII activity.

Plants long-term acclimation to the cold survival is intensely correlated with the recovery of photosynthesis at low temperatures (Stitt and Hurry, 2002) and the maintenance of soluble carbohydrate reserves (Olien and Clark, 1993). Considering the Pn reduction due to cold stress, our results suggest that one night at 0 or -1°C triggered stomatal limitation of photosynthesis in *A. thaliana*, while -3°C treatment led to a non-stomatal limitation. Wilkinson et al. (2001) showed that *A. thaliana* could respond to cold by stomatal closure. However, the pattern of photosynthesis limitation may depend on temperatures as described in grapevine leaves (Flexas et al., 1999) and inflorescences (Sawicki et al., 2012). Freezing temperatures could inhibit the Calvin cycle through loss of RuBisCO activity or reduction of ribulose-1,5-bisphosphate regeneration rate, resulting in a decrease of Pn without Ci decrease (Allen and Ort, 2001). In the present study, three nights at -1°C slightly reduced RbcS contents without modifying gene expressions confirming literatures that have reported that RuBisCO protein is mainly regulated by *RbcS* genes. In addition, in accordance with our results, Abbott and Bogorad (1987), Prioul and Reyss (1988), Piechulla (1989), have shown the low responsiveness of *RbcL* mRNA to environmental factors. The expression of chloroplast-encoded genes, as *RbcL*, is more likely regulated at the post-transcriptional level (Mullet, 1988). Strand et al. (1999) and Hurry et al. (2000) noted that the recovery of photosynthesis and the development of freezing tolerance are strongly correlated with a reprogramming of carbon metabolism.

In this study, all three *CBF* genes were overexpressed without modification of *ICE1* expression at the end of three cold nights or days. It has been shown that cold induction of *CBF* genes is under the control of a complex network involving *ICE1* but also other transcription factors belonging to the MYB family (Theocharis et al., 2012b). In *Arabidopsis, CBF1, 2*, and *3* are involved in the regulation of *COR* gene expression and cold tolerance (Thomashow, 1999, 2010), and regulated by promoters, such as *ICE1*. Additionally, expression of the three *CBF* genes in *Arabidopsis* could also be influenced by light quality, circadian rhythm and photoperiod (Fowler et al., 2005; Lee and Thomashow, 2012; Maibam et al., 2013). In *Arabidopsis*, the four major COR genes, COR6.6/KIN2, COR15A, COR47/RD17, and COR78/RD29A, encode highly hydrophilic and boiling-stable proteins that play a role in the stabilization of membranes and proteins under freeze-induced dehydration conditions (Thomashow, 1999). Our results showed that expression of *COR15a* and *COR78* was increased only after three cold nights. Regulation of *CBF* target gene *COR78* is slightly impacted by the circadian rhythm (Fowler et al., 2005; Medina et al., 2011). In *A. thaliana*, overexpression of CBF1 and CBF3 activates COR gene expression and enhances freezing tolerance (Liu et al., 1998; Maruyama et al., 2004).

### Impact of *Bp PsJN* during a Cold Stress

Beneficial effects triggered by PGPR colonization against cold are associated to photosynthesis, carbohydrates and related metabolites (Ait Barka et al., 2006; Fernandez et al., 2012a,b). In this study, *Bp PsJN* inoculation did not affect PSII activity and gas exchange during and after one cold night at 0 or -1°C. On the opposite, the presence of *Bp PsJN* increased Pn after one night at -3°C. Considering the lower Ci, in inoculated plants, we suggested that *Bp PsJN* enhances photosynthesis by non-stomatal mechanisms after -3°C exposure. In grapevine, the presence of *Bp PsJN* reduces impact of chilling on Pn (Ait Barka et al., 2006; Fernandez et al., 2012a) *via* a non-stomatal dependent pattern (Fernandez et al., 2012a).

Bacteria *Bp PsJN* affects photosynthetic pigment accumulation and *RbcL* gene and protein accumulation after freezing treatment. Beneficial effect was shown on chlorophyll contents after three freezing nights whereas a significant reduction of *RbcL* expression and protein content was visible upon cold night exposure in bacterized plants. In contrast, Fernandez et al. (2012a) have shown that *Bp PsJN* presence did not modified pigment concentration (chlorophyll and carotenoid) in grapevine after 5 cold days.

Poupin et al. (2013) reported that *Bp PsJN* inoculation modified several gene regulations in *Arabidopsis*, including genes involved in defence or in biotic or abiotic stimulus responses. In grapevine, *Bp PsJN* could impact *CBF* gene expression to enhance plant cold tolerance (Theocharis et al., 2012a). Here, the expression of *COR78* gene was decreased whereas the expression of *CBF* or *ICE* genes were not modified by the presence of *Bp PsJN*. Similarly, Xin and Browse (1998) demonstrated that *esk1* (*eskimo1*) mutant exhibits freezing tolerance without over-expression of *CBF* genes. Furthermore, *sfr* (*sensitive to freezing*) mutants are severely compromised in their ability to develop freezing tolerance but the regulation of *CBF*/*DREB* genes is unaffected (Knight et al., 1999). These results suggest that a pathway distinct from the CBF cold response pathway could be involved in freezing tolerance induced by *Bp PsJN*.

It is interesting to note that only expression of *COR78* was repressed during cold treatment in *Bp PsJN*-bacterized plants compared with mock-inoculated plants, while expression of *COR15a* was not impacted (only after night stress) despite that both have the same pathway. Hajela et al. (1990) reported a primary regulation at both the transcriptional (COR15a) and the post-transcriptional (COR78) levels. Similarly, differences in the tissues specificity of the promoters of these genes were reported. The *COR15a* promoter fused to GUS reporter gene is expressed in anthers of non-acclimated plant (Baker et al., 1994) while the *COR78* promoter is not (Horvath et al., 1993). Furthermore, in cold-acclimated plants, the COR15a promoter is expressed in all flower parts except the ovary (Baker et al., 1994) while the *COR78* promoter is expressed in the sepals, petals and anther filaments (Horvath et al., 1993). These funding have interesting implications regarding potential *cis*-acting cold-regulatory elements. Consequently, there must either be a family of *cis*-acting cold-regulatory elements, with each member having a different tissues specificity, and/or the *cis*-acting cold-regulatory elements interacts with other tissue specific elements to provide the cold regulation pattern of gene expression.

We showed that *Bp PsJN* induced a cell wall strengthening, similar to that observed following cold exposure, after either night or day-cold treatments. Previous works have shown that cold exposure increases cell wall strength and decreases the pore-size of the cell wall (Rajashekar and Lafta, 1996). Cold stress induces accumulation of cell wall components including pectin and non-cellulosic components (Kubacka-Zebalska and Kacperska, 1999). It has been proposed that cell wall rigidity may be an important factor in cell resistance to cold stress (Rajashekar and Lafta, 1996). It was also suggested that cell wall architecture is important in plant resistance to abiotic stress (Hamann, 2012; Le Gall et al., 2015) and essential in cold-stress sensing and signal transduction (Seifert and Blaukopf, 2010). We may thus hypothesize that *Bp PsJN* could prevent freezing damages and cell collapse by inducing a cell wall strengthening. Further, the potential implication of COR genes in the stabilization of membranes and proteins under freeze-induced dehydration conditions (Thomashow, 1999) may partly explain the maintenance of cell integrity observed in the bacterized plant under low temperatures.

In the present work, we have studied the impact of the presence of *PsJN* on the tolerance of *A. thaliana* to low temperatures. The issue of the results indicates a strengthening of cell wall as response to the presence of *PsJN* compared to non-bacterized plants. Moreover, after a night stress the bacteria led to better photosynthetic pigment content. We might consequently suggest that the strain *Bp PsJN* alleviate cold damages by acting on both photosynthesis and cell morphology.

## Author Contributions

FS, CJ, EB, SD-C, and NV-G designed the research. FS, CJ, SV, JM, SD-C, and NV-G carried out the experiments and analysis/interpretation of data. FS, CJ, EB, CC, SD-C, and NV-G wrote the manuscript with contributions and discussion from all of the co-authors. All authors have given approval to the final version of the manuscript.

## Conflict of Interest Statement

The authors declare that the research was conducted in the absence of any commercial or financial relationships that could be construed as a potential conflict of interest.

## Abbreviations

*BP, PsJN*: *Burkholderia phytofirmans* strain *PsJN*
CBF: C-repeat binding factor
Ci: intercellular CO_2_ concentration
COR: cold-responsive
E: transpiration rate
ETR: electron transport rate
Fv/Fm: maximum efficiency of PSII
g_s_: stomatal conductance
ICE: inducer of CBF expression
PGPR: plant-growth-promoting rhizobacteria
Pn: net photosynthesis
PSII: photosystem II
RbcL: RuBisCO large subunit
RbcS: RuBisCO small subunit
RuBisCO: ribulose-1,5-bisphosphate carboxylase/oxygenase
TEM: transmission electron microscopy
ΦPSII: effective quantum yield of PSII
